# Correlating Various Clinical Outcomes Associated with Motor Vehicle Collision-Related Trauma

**DOI:** 10.3390/healthcare13182314

**Published:** 2025-09-16

**Authors:** Bharti Sharma, Luka Stepanovic, Sittha Cheerasarn, Samantha R. Kiernan, George Agriantonis, Navin D. Bhatia, Shalini Arora, Zahra Shafaee, Kate Twelker, Jennifer Whittington

**Affiliations:** 1 Department of Surgery, NYC Health and Hospitals—Elmhurst, New York, NY 11373, USA; lukastepanovic01@gmail.com (L.S.); cheerass@nychhc.org (S.C.); agriantg@nychhc.org (G.A.); bhatian1@nychhc.org (N.D.B.); arorash@nychhc.org (S.A.); shafaeez1@nychhc.org (Z.S.); twelkerk1@nychhc.org (K.T.); harrisj20@nychhc.org (J.W.); 2 Department of Surgery, Icahn School of Medicine at Mount Sinai, New York, NY 10029, USA; 3 Department of Medicine, Touro College of Osteopathic Medicine–Harlem, New York, NY 10027, USA; skiernan@student.touro.edu

**Keywords:** trauma, motor vehicle collision, clinical outcomes, payor disposition, severity, length of stay

## Abstract

**Objectives:** Despite the implementation of additional safety measures, motor vehicle collisions (MVCs) still result in significant injuries and fatalities. This study aims to explore the severity of these injuries and the length of hospital stays (LOS) following MVCs. Furthermore, this study will assess how helmet use and alcohol influence trauma outcomes. **Methods:** This retrospective study from a single center includes 604 patients from 1 January 2016, to 31 December 2024. Patients were identified based on the Abbreviated Injury Scale (AIS) body regions. Descriptive statistics and ANOVA were performed on helmet use and blood alcohol concentration, with significance set at *p* < 0.01. **Results:** Mean LOS at the hospital (H) was 13 days, 10.53 h in the ED, and 113.32 h in the ICU. In total, 74.5% of patients were male and 25.5% were female. The mean injury severity score (ISS) was 22.58, with 99.83% representing blunt trauma. The majority of patients (94.21%) arrived with signs of life, with 50.99% patients discharged to home or self-care (routine discharge). A noticeable trend following 2020 showed an increase in ED discharges, and thus ED admissions, compared to years before 2020. Helmet use showed a non-significant trend toward reduced ISS and length of stay. ETOH level and primary payor source were not significantly associated with outcome variables in regression models, though patterns suggest a potential relationship between payor source and ED discharge disposition. **Conclusions**: This study identifies important clinical trends that merit further investigation. Helmet use may be associated with reduced injury severity and shorter hospital stays, while differences in primary payor source suggest disparities in ED discharge outcomes. These findings underscore the need for further research on payor disposition, helmet use, and ETOH level in MVCs.

## 1. Introduction

Motor vehicles are an integral part of transportation in the United States. Cars, motorcycles, trucks, and scooters are all classified as motor vehicles. With the number of miles traveled increasing with registered vehicles as the population grows, motor vehicle collisions (MVCs) are a serious concern. Even though MVC fatalities have decreased in the United States over the past few decades, there was a 10% increase in 2021 compared to 2019, and numbers have still not decreased to their pre-pandemic levels [1]. Over the last decade, roadway fatalities have, on average, increased. In 2021, 43,320 people were killed in MVCs in the United States, which is the highest number since 2005 [1]. Moreover, MVCs also have financial implications, which include wage and productivity loss, medical expenses, administrative expenses, motor vehicle property damage, and employer costs. In the United States in 2023, these motor vehicle injury costs were USD 513.8 billion [2]. Due to these risks, technology such as seatbelts, helmets, and policies such as reducing the blood alcohol concentration (BAC) limit have been implemented.

Helmet use, particularly in bicycle and motorcycle crashes, has been proven to be effective in reducing the severity of head injuries. In a 2016 study from Hawai’i, non-helmeted motorcycle and moped riders were nearly three times more likely to die [3]. Lack of helmet use is strongly associated with higher Abbreviated Injury Scale (AIS) scores for head trauma, which measure injury severity from 1 (minor) to 6 (unsurvivable). Higher AIS head scores are predictive of worse outcomes, including long-term disability or death. Overall, helmet use can reduce the risk of head injury by up to 74% in MVCs and halve the risk of severe traumatic brain injury and death [4,5,6,7]. However, the decrease in fatality rates is reduced with the looseness of helmet laws from state to state [8]. New York has some of the strictest laws for motorcyclists, requiring all motorcycle drivers and passengers to wear a helmet, but bicyclists are not required to wear a helmet if they are over the age of 14 [9,10].

Alcohol intoxication is also a major risk factor in MVCs. Elevated BAC has a well-established association with slower reaction times, impaired judgment, and decreased coordination, all of which significantly increase the likelihood of crashes and injury severity [11]. According to the National Highway Traffic Safety Administration, BAC levels as low as 0.02% can cause drivers to begin to experience altered visual functions, reduced ability to multitask, and some loss of judgment [11]. At a 0.05% BAC, drivers can experience reduced ability to track moving objects, difficulty steering, and, most importantly, a reduced response to emergencies [11]. MVCs that involve patients who test with a BAC above the legal limit sustain a higher injury severity score and mortality than negative patients, and above-the-limit patients are more likely to be found at fault in the crash [12,13,14]. Interestingly, patients with high BAC levels have increased odds of being non-helmeted, and due to the compounding effect, can be up to 3 times more likely to die than BAC-negative patients [12,14,15,16].

In addition to clinical outcomes, the financial and systemic implications of MVCs, as mentioned before, are important and are reflected in hospital payor disposition. Insurance status and discharge destinations can offer insight into broader disparities in healthcare access, long-term support, and socioeconomic burden. However, this remains an area for further research.

Given the established relationships between helmet use, blood alcohol concentration, and injury severity in motor vehicle collisions, further investigation is warranted to expand on how these variables can influence outcomes such as hospital length of stay, injury severity score, and mortality. The primary aim of this study is to provide a comprehensive study on patients involved in MVCs relating to helmet use, BAC, injury severity score, and hospital length of stay. A secondary aim is to analyze ED disposition by the factor of primary payor source. This study can enhance the current understanding of risk factors in trauma care relating to MVCs and contribute to improving outcomes for patients involved in MVCs.

## 2. Methods

This is a single-center, retrospective review conducted at a Level 1 trauma center in New York City from 1 January 2016 to 31 December 2024. Only patients with motor vehicle injuries were included. Patient data were requested from the trauma registry at our facility (Elmhurst Hospital Center). Our center’s trauma registry utilizes NTRACS software (ver.12.30.24) (American College of Surgeons National Trauma Registry System, https://www.facs.org/quality-programs/ (accessed on 2 March 2025). Extracted data included demographics, identified based on the cause of injury, helmet use, primary mechanisms (lCD9 or lCD10 E-Code, injury diagnosis codes), trauma type, length of stay (LOS) at the emergency department (ED), intensive care unit (ICU), and hospital (H), the injury severity score (ISS, a standardized scoring system used to assess the overall severity for trauma patients with multiple injuries), and the Abbreviated Injury Severity (AIS, measures specific injuries on a scale from 1, minor, to 6, unsurvivable) head score. ETOH levels were grouped to avoid oversights into the following categories: undetectable (0), low (1–79), moderate (80–199), high (200–299), extreme (>300). The thresholds for low and moderate ETOH levels were based on the legal limit for operating a motor vehicle in the state of New York. Pearson chi-square tests were conducted to test the association between certain categorical variables. The Kruskal–Wallis test and Mann–Whitney U with post hoc pairwise comparisons using the Bonferroni correction were used to test whether various scores and lengths of stay differed between different demographics and groupings, such as the ETOH level. Additionally, data regarding LOS in the ED, ICU, and H, trauma type, and type of mortality, means, and percentages were analyzed. For payor type, chi-square tests and Fisher tests were used. All analyses were conducted in SPSS Version 31.0.0.0.

## 3. Results

During 9 years, 604 patients were classified as MVCs. Demographic details can be found in Table 1 (ethnicity/gender/count/percentage of total patients/average age). The majority of patients were male (74.5%), while females comprised 25.5% of the study population. The average age of all patients was 42.2 years, with females tending to be older on average (49.0 years) compared to males (39.9 years).

In terms of ethnic distribution, the largest group was “other” (males 49.5%, females 12.4%), with a significant proportion identifying as Hispanic origin (males 36.42%, females 7.95%). White patients made up the next highest cohort (males 8.8%, females 5.6%), followed by Asian patients (males 7.9%, females 5.0%). Black patients represented a smaller portion of the sample (males 3.97%, females 0.5%). A minor percentage of patients were identified as Native Hawaiian or other Pacific Islander (0.33% for both males and females).

**Figure 1:**
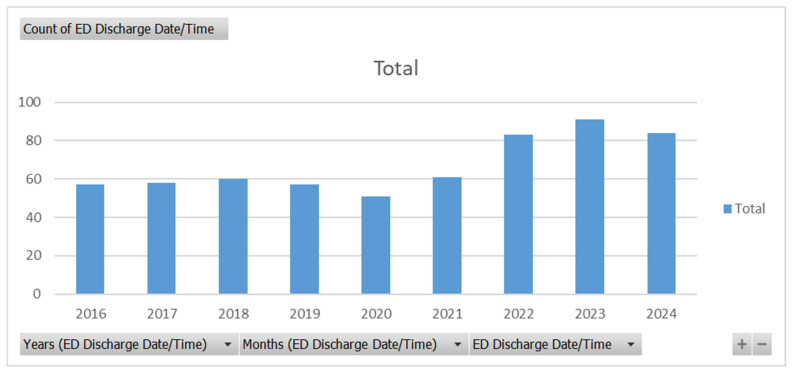
Count of ED discharge patients related to MVCs from 2016 to 2024.

The number of patients discharged from the ED is shown in Figure 1. Out of a total of 602 patients, the number of patients discharged from the ED has increased in the previous years compared to before 2021. There was a drop in ED discharges in 2020, which might suggest the effect COVID-19 had on reducing motor vehicle travel. However, before COVID-19, ED discharge patients were much less than in the years after COVID-19. This might suggest a shift in injury severity, practices in the ED, or an increase in vehicle traffic and unsafe motor vehicle travel.

Injury severity and hospital length of stay data are shown in Table 2. Among the cohort, a total of 7799 hospital days were recorded, with an average LOS of 13.04 days per patient. The majority of time spent in the hospital was distributed as follows: 60.53% in other inpatient units (113,599.28 h), 36.11% in the ICU (67,768.32 h), and only 3.35% in the emergency department (ED) (6295.25 h). This suggests that most patients required care beyond the ED, with a substantial portion requiring intensive care services. Patients presented with an average initial Glasgow Coma Scale (GCS) of 11.25, indicating a moderately impaired level of consciousness at presentation. The average injury severity score (ISS) was 22.58, consistent with severe trauma. The AIS head score averaged 3.71, indicating that head injuries were common and generally moderate to severe. Out of 598 total traumatic injuries, 99.83% were blunt trauma, while only 0.17% were penetrating injuries, reflecting the predominance of MVCs and similar blunt-force mechanisms.

Mortality and discharge dispositions are shown in Table 3. Among the 604 patients included in this analysis, 35 patients (5.79%) arrived at the hospital with no signs of life. Of these, 34 (5.63%) were confirmed deceased as full code, and 1 patient (0.17%) had an unknown cause of death. The majority of patients, 569 (94.21%), arrived with signs of life. Among this group, 63 patients (10.42%) died during hospitalization, with 44 (7.28%) dying as full code, 13 (2.15%) following the withdrawal of care, and 6 (0.99%) categorized as DNR/DNI, where care was not initiated. Seventeen patients (2.81%) met brain death criteria. Regarding discharge disposition, 308 patients (50.99%) were discharged home or to self-care, while 20 (3.31%) required home services. A small number of patients were discharged to alternative facilities or care pathways, including 33 (5.46%) to inpatient rehabilitation, 19 (3.15%) to sub-acute inpatient rehabilitation, and 44 (7.28%) to traumatic brain rehabilitation centers. Additionally, 13 patients (2.15%) were transferred to skilled nursing facilities, 14 (2.32%) to other acute care hospital emergency departments, and 11 (1.82%) to other acute care hospital inpatient units. Fewer patients were discharged to hospice, inpatient psychiatric care, other healthcare facilities, correctional facilities, or specialized rehabilitation centers. These findings highlight that while the majority of patients survived their injuries, a significant proportion required specialized or prolonged care, and mortality remained notable among those arriving with or without signs of life.

Of the 604 total MVC patients, 261 were involved in a crash where a helmet would be commonly worn (motorcyclists, bicyclists, etc.). A comparative analysis using Mann–Whitney U tests was conducted to examine the relationship between helmet use and the outcome of interest across 261 observations (Table 4a). The model demonstrated significance values > 0.05 for each length of stay variable, indicating that helmet use was not statistically significant in patients’ length of stay in any department or overall. Mann–Whitney U and Wilcoxon W analysis reported asymptomatic two-tailed significance values of 0.194 for ED length of stay, 0.701 for ICU length of stay, and 0.832 for hospital length of stay. These findings suggest that helmet use, within this sample, did not significantly impact the measured outcome.

A comparative analysis using the Mann–Whitney U test was conducted to examine the relationship between helmet use and the outcome of interest across 261 observations (Table 4b). The model demonstrated significance values > 0.05 for each length of stay variable, indicating that helmet use was not statistically significant in patients’ trauma scores. Mann–Whitney U and Wilcoxon W analysis reported asymptomatic two-tailed significance values of 0.401 for ISS, 0.155 for initial GCS total scores, and 0.344 for AIS head scores. These findings suggest that helmet use, within this sample, did not significantly impact the measured outcome.

A chi-square analysis was performed to examine the relationship between helmet use and mortality in motor vehicle crashes (Table 4c). The model explained very little of the variance in mortality, with a two-sided *p* = 0.522 and one-sided *p* = 0.316, indicating that helmet use was not a meaningful predictor of mortality in this sample of 261 observations. Both Phi and Cramer’s V values were 0.5, with Fisher’s exact test confirming the absence of any statistical significance in the use of helmets regarding mortality in patients who were required to use them.

Kruskal–Wallis analysis was conducted to assess the association between ETOH level and ISS, AIS head, and total GCS in 500 cases (Table 5). The model explained a negligible amount of variance in ISS (*p* = 0.079), indicating that ETOH level did not meaningfully predict injury severity, as well as a low statistical significance (*p* = 0.165) for AIS head scores. For total GCS scores, however, Kruskal–Wallis tests indicated rejection of the null hypothesis (*p* < 0.001). Bonferroni correction was used during pairwise comparison of total GCS scores across ETOH groups and came back with significant values between the following ETOH level groupings: extreme–moderate (*p* = 0.01), extreme–undetectable (*p* = 0.001). This indicates that ETOH levels had a significant association with total GCS scores when comparing extreme levels to moderate and undetectable levels.

Kruskal–Wallis analysis was conducted to evaluate the relationship between ETOH level and LOS in 498 patients (Table 5b). The model demonstrated low significance values for emergency department length of stay (ED LOS, *p* = 0.224), intensive care unit length of stay (ICU LOS, *p* = 0.249), and overall hospital length of stay (hospital LOS, *p* = 0.102). There was no significant correlation demonstrated from nonparametric Kruskal–Wallis analysis between ETOH levels and length of stay in any department.

Chi-square analysis was performed to examine the relationship between ETOH level and the outcome variable in a sample of 499 observations (Table 5c). The model resulted in statistically significant values with two-sided Pearson chi-square (*p* = 0.005), and furthermore with Fisher’s exact test (*p* = 0.013) and Monte Carlo significance (*p* = 0.007). Additionally, Cramer’s V (*p* = 0.005) was confirmed. All values led to the conclusion that ETOH groups had a significant correlation with mortality in these cases.

Chi-square analysis was performed to examine the relationship between demographic characteristics and mortality in a sample of 603 observations (Table 6a,b). In regard to the comparison of age groups to mortality, the model resulted in statistically significant values with Pearson chi-square (*p* < 0.001), and furthermore with Fisher’s exact test (*p* = 0.001) and Monte Carlo significance (*p* = 0.001). However, Cramer’s V (*p* < 0.001) indicated that the strength of the association was incredibly weak. The analyses led to the conclusion that age groups had a weak but statistically significant association with mortality in these cases.

In the case of comparative chi-square analysis of gender and mortality, the results differed, with Pearson chi-square being insignificant (*p* = 0.532), as well as Fisher’s exact test (*p* = 0.535). The conclusion is that gender had no statistically significant correlation with mortality in this cohort.

A final comparison was run to analyze race and mortality. Values from Pearson chi-square (*p* = 0.026) and a Monte Carlo estimate (*p* = 0.023) indicated a statistically significant correlation between race and mortality. Phi and Cramer’s V values (*p* = 0.026) noted that, while present, the correlation was not very strong.

Kruskal–Wallis analysis was performed to examine the correlation between age groups and length of stay in the ED, ICU, and hospital overall (Table 7). All results indicated that there was a statistically significant correlation (*p* < 0.05) between age groups and their length of stay in each department, as well as overall in the hospital. Pairwise comparison was made with Bonferroni correction, with the following results: In each department and overall (ED LOS, ICU LOS, and hospital LOS), adjusted significance rates indicated that there was a statistically significant correlation (*p* < 0.05) when ages 0–15 were compared to the other categories. The adjusted significance rates for the other age groups were all insignificant (*p* > 0.05). This led to the conclusion that the youngest group (0 < age < 15) spent significantly less time in the ED, ICU, and hospital overall when compared to each of the other groups.

Additional Kruskal–Wallis and Mann–Whitney U analyses were performed individually to establish any significance between age groups, gender, and race with length of stay in any department. All tests resulted in retaining the null hypothesis (*p* > 0.05), leading to the conclusion that there were no further statistically significant correlations between any of the demographic groups listed and LOS or trauma scores (total GCS, AIS, and ISS).

Separate analyses were performed using Kruskal–Wallis tests to examine AIS head scores and hospital discharge dispositions (Table 8). Initial results reported there was a statistically significant correlation (*p* < 0.001); thus, post hoc pairwise comparison with Bonferroni correction was examined as well. The following discharge disposition comparisons were significant: routine discharge/rehabilitation required (*p* < 0.001), routine discharge/died (*p* < 0.001), other/died (*p* < 0.001). All other pairs were not significant after adjustment. Interpretation concluded that AIS head scores were significantly higher in groups that died or required rehabilitation than in others.

A contingency analysis was performed to examine the association between primary payor source and hospital discharge disposition in 603 patients (Table 9 and Table 10). The overall relationship between payor source and discharge disposition was found to be statistically significant. The chi-square test revealed a significant association between payor type and discharge disposition (*p* < 0.001). However, 66.3% of the cells had expected counts less than 5, indicating that the assumptions for the chi-square test were not fully met. To account for this, Fisher’s exact test using Monte Carlo simulation was conducted, which also showed a statistically significant association (*p* < 0.001). The strength of association was small to moderate, as reflected by Cramer’s V = 0.228. These findings suggest that a patient’s primary payor source is significantly associated with their discharge disposition, though the strength of this association is limited.

## 4. Discussion

This study provides valuable insight into MVCs over 9 years, focusing on the demographics, ED disposition, length of stay, helmet use effectiveness, BACs, and payor disposition. Similar to other study cohorts, there was a predominantly male population with an average age in the 30s and early 40s [3,4,5,6,17,18]. Our cohort had a notable proportion of patients identifying as Hispanic. This could be a representation of the community that Elmhurst Hospital is located in, and may not be generalized to other populations. Additionally, similar to the data from the U.S. Department of Transportation, there was a peak of fatalities, and thus ED admissions and discharges, in 2021, and this number has been decreasing; however, it is still above the number before 2020 [1]. This could reflect a negative shift in motor vehicle practices and safety after COVID-19.

Our cohort had an average H LOS of 13.04 days, with most time spent in inpatient and ICU settings. This LOS is longer than some published averages, such as 3.1 days reported in another trauma cohort, though differences in injury severity and trauma system resources may explain this difference [19]. Studies consistently show that higher injury severity scores (ISSs) and more severe injuries (e.g., ISS ≥ 12 or LOS ≥ 7 days) are associated with longer hospital stays and poorer long-term outcomes [5,20,21]. In particular, patients with severe injuries (ISS ≥ 12) have a significantly longer LOS and worse physical health at 12 months post-injury [20]. Our data of ISS = 22.6 aligns with these findings, thus supporting that injury severity positively correlates with prolonged hospitalization [20]. However, there is limited research that delves into ED length of stay and ICU length of stay. Our cohort also had an AIS head score of 3.71, indicating a predominance of moderate-to-severe head injuries. We did not delve into the consequences of this score; however, the literature does indicate that higher AIS head scores are strongly associated with increased mortality and a greater likelihood of discharge to rehabilitation [22]. Furthermore, AIS scores > 2 have been found to have a link to non-helmeted groups, which could indicate that a majority of our population did not have helmets [5]. Our cohort does model the consensus that blunt trauma accounts for nearly all cases in MVCs [19].

Helmeted individuals in our dataset appeared to have shorter hospital stays and lower mortality rates compared to non-helmeted individuals, suggesting a potential protective effect. However, the regression analyses for helmet use found no statistically significant association between helmet use and length of stay, injury severity score, or mortality. This contrasts with most research that shows that helmet use is strongly associated with lower mortality and short hospital stays [5,8,20,21]. For example, helmet use has been linked to a 56% decrease in mortality and an 8% reduction in hospital charges, and universal helmet laws are associated with a 36–45% decline in motorcycle crash mortality [8,21]. However, some studies indicate that helmet type might not be a predictor of injury severity, and the protective effect of helmets may be less pronounced in certain groups and settings [18]. The null findings may reflect sample size, local helmet compliance, or helmet quality used. Additionally, our study did not differentiate between detailed mechanisms of transport where a helmet would commonly be used (i.e., motorcycle crashes, bicycle crashes, e-bike crashes, riders struck by vehicles, etc.), which may provide further insight and statistical significance. Further studies should investigate the specific breakdown of injuries by mechanism for helmet users to provide further guidance on public health measures that might be taken to mitigate risk.

The regression analyses for BAC also found no significant association between BAC and injury severity, length of stay, or mortality. Previous research indicates that alcohol intoxication increases the risk of severe injury and death, and is also associated with helmet non-use [14,22,23,24]. In Wiratama et al., alcohol-involved riders were 9.47 times more likely to sustain fatal injuries, and alcohol-involved and non-helmeted riders were 18 times more likely [22]. This indicates the dangerous nature of alcohol and MVCs; however, Li et al. have indicated that alcohol intoxication had a high risk for mortality but was not statistically significant in their cohort [4]. This suggests that other factors, such as injury mechanism and hospital care, may mediate outcomes. It is also possible that further dividing our cohort by vehicle type (car, motorcycle, bicycle, etc.) may provide more insights. Additionally, our observed mortality rate (5.8% on arrival with no signs of life, 7.2% died as full code) is consistent with other studies, where mortality rates range from 5% to 12% [4,18].

Our study did find a statistically significant association between payor source and ED disposition, though the strength was modest. The literature on payor status and MVC trauma outcomes is limited, but differences in access to care and discharge disposition by insurance type have been suggested as potential contributors to disparities in trauma outcomes [17]. A few studies found that Medicaid beneficiaries had a significantly longer LOS compared to all other patients [25,26], suggesting that there are specific factors for Medicaid patients that lead to a need for increased care. Further studies showed that uninsured trauma patients had higher mortality rates than insured patients [27,28] and had worse long-term functional outcomes [27]. These findings show the potential burden that Medicaid and uninsured patients have on hospital systems, and the increased morbidity and mortality of these patients. Prompting the need for further research to provide more specific public health solutions.

Compared with the prior literature, our study contributes by simultaneously analyzing helmet use, alcohol intoxication, hospital course, and insurance status in a large, urban trauma cohort. This comprehensive approach highlights that while helmet use and BAC did not independently predict severity or mortality, payor status did emerge as a significant factor in disposition, underscoring the role of systemic inequities. These findings extend the discussion beyond prevention alone and emphasize the intersection between trauma care and health system structure.

Interpretation of these findings must also be placed in the context of the U.S. healthcare system. Trauma care in the United States is regionalized, with Level 1 trauma centers serving as referral hubs equipped with advanced ICU, surgical, and rehabilitation services. Because access to post-acute care is often mediated by insurance type, discharge planning and long-term outcomes are shaped not only by clinical needs but also by coverage and socioeconomic barriers.

Clinically, our results have several implications. The lack of statistical significance for helmet use in regression analyses should not discourage helmet promotion but instead points toward the need for more in-depth data collection on helmet quality, crash mechanism, and compliance. Finally, the link between payor source and disposition highlights a role for trauma clinicians in advocating for equitable rehabilitation access and strengthening hospital-community transitions, particularly for Medicaid and uninsured populations. Together, these implications support a dual strategy: reinforce prevention through helmet and alcohol harm-reduction policies, while addressing systemic barriers that uniquely influence trauma care outcomes in the U.S.

### 4.1. Strengths

This study provides a comprehensive, multi-faceted analysis of MVCs over 9 years at a major urban trauma center, with a focus on injury severity, hospital and ICU LOS, helmet use, BAC, and primary payor status. This study adds valuable insight into payor type by ED disposition, an area that is underrepresented in the current trauma literature. The inclusion of variables such as ISS and LOS allow for a more nuanced understanding of hospital resource utilization. The alignment of our ISS and LOS data with previously published findings also supports the internal validity of the dataset. Moreover, our sample reflects the trauma burden at Elmhurst Hospital and a high proportion of Hispanic and underinsured patients.

### 4.2. Limitations

Despite its strengths, this study has limitations. Most notably, many of the regression models yielded non-significant *p*-values and very low R^2^ values, indicating that the independent variables studied (helmet use and BAC) explained very little of the variability in trauma outcomes. This may be due to confounding variables not accounted for in the model, insufficient sample sizes within subgroups, or how variables were categorized. For example, BAC was treated as a continuous variable, instead of analyzing BAC in categorical ranges (e.g., 0, 0.01–0.08, >0.08), which may have revealed significant relationships. Similarly, there is a lack of interaction analysis between BAC and helmet use, despite prior research indicating that there might be a compounding effect. Additionally, this was a single-center, retrospective study, which limits its generalizability. The cohort largely reflects the demographic profile of the community surrounding Elmhurst Hospital, with a significant proportion identifying as Hispanic. While this provides important insight into local trends, it may not be representative of trauma populations in other geographic or demographic contexts. This study was also conducted within the American healthcare system, and so conclusions reached regarding payor status may not be applicable internationally. Additionally, while the analysis captured overall LOS, ICU LOS, and ED disposition, it did not examine the consequences of high AIS scores. The quality and type of helmets used would have been beneficial to add to the dataset.

## 5. Conclusions

This study provides a comprehensive 9-year overview of MVC trauma care at a Level 1 trauma center in New York City, highlighting patterns in demographics, injury severity, hospital length of stay, helmet use, BAC, and primary payor disposition. Consistent with prior research, the cohort was predominantly male and middle-aged, with a substantial proportion identifying as Hispanic, likely reflecting the community. While helmeted individuals appeared to experience better outcomes, including lower mortality and shorter hospital stays, these associations did not reach statistical significance. Similarly, BAC levels showed no significant correlation with injury severity or outcomes, challenging assumptions about alcohol-related trauma. These null findings may be due to limited sample sizes, data categorization, or unmeasured variables such as helmet quality or injury mechanism. Most importantly, the study found a statistically significant association between primary payor type and emergency department disposition, signaling potential disparities in trauma outcomes that could indicate further research. This study not only reflects local trauma care trends but also contributes to broader discussions on public health, safety policies, and healthcare equity in urban trauma systems.

## Figures and Tables

**Table 1:** Demographic information of patients who encountered motor vehicle crashes.

Demographics	Male		Female	
	Count (%)	Average Age	Count (%)	Average Age
Asian	48 (7.95)	45.22	30 (4.97)	56.64
Black	24 (3.97)	39.89	3 (0.50)	28.78
Native Hawaiian or Other Pacific Islander	2 (0.33)	32.53	2 (0.33)	50.68
White	53 (8.77)	43.61	34 (5.63)	53.05
Other	299 (49.50)	38.24	75 (12.42)	45.41
Unknown	24 (3.97)	42.13	10 (1.66)	45.17
Hispanic Origin	220 (36.42)	38.16	48 (7.95)	50.24
Non-Hispanic Origin	195 (32.28)	42.16	90 (14.90)	48.87
Total	450 (74.50)	39.89	154 (25.50)	49.01

**Table 2:** This table represents the length of stay in the ED, ICU, and other units in the hospital. The average AIS head score and ISS score are reflected.

Injury Severity/LOS	Average
Hospital days	13.04
Hours	104.61
ED hours	10.53
ICU hours	113.32
Other hours	189.97
ED/hospital initial GCS	11.25
ISS	22.58
AIS head	3.71

**Table 3:** Patient mortality and discharge disposition following motor vehicle collisions.

Mortality	Count of Patients	%
Arrived with NO signs of life	35	5.79%
Died as full code *	34	5.63%
Died unknown	1	0.17%
Arrived with signs of life	569	94.21%
Blank/unknown	1	0.17%
Died after withdrawal of care	13	2.15%
Died as full code *	44	7.28%
Died with care, not begun DNR/DNI	6	0.99%
Discharged to home or self-care (routine discharge)	308	50.99%
Home with services	20	3.31%
Homeless/shelter	1	0.17%
Hospice	2	0.33%
Inpatient psych care	1	0.17%
Inpatient rehabilitation	33	5.46%
Left AMA	10	1.66%
Met brain death criteria	17	2.81%
New placement at a skilled nursing facility	13	2.15%
Other acute care hospital emergency department	14	2.32%
Other acute care hospital inpatient	11	1.82%
Other health care facility	5	0.83%
Police custody/jail/prison	5	0.83%
Spinal cord injury rehabilitation	2	0.33%
Sub-acute inpatient rehabilitation	19	3.15%
Traumatic brain rehabilitation	44	7.28%

* Note: “Full code” refers to the United States shorthand for patients designated for full resuscitation (i.e., CPR and advanced life-sustaining measures).

**Table 4:** (**a**) The relationship between helmet use and the outcome of interest across 261 observations using Mann–Whitney U analysis. (**b**) The relationship between helmet use and the outcome of interest across 261 observations using Mann–Whitney U analysis: helmet use and trauma scores. (**c**) The relationship between helmet use and mortality in motor vehicle crashes using chi-square analysis.

**(a)**					
	**Helmet**		**N**	**Mean Rank**	**Sum of Ranks**
ED Length of Stay	Required— Not Worn		190	134.71	25,594.50
	Required—Worn		71	121.08	8596.50
	Total		261		
ICU Length of Stay	Required— Not Worn		190	132.06	25,092.00
	Required—Worn		71	128.15	9099.00
	Total		261		
Hospital Length of Stay	Required— Not Worn		190	131.10	24,908.50
	Required—Worn		70	128.88	9021.50
	Total		260		
	**ED Length of Stay**		**ICU Length of Stay**		**Hospital Length of Stay**
Mann–Whitney U	604.500		6543.000		6536.500
Wilcoxon W	8596.500		9099.000		9021.500
Z	−1.298		−0.385		−0.212
Asymp. Sig. (2-tailed)	0.194		0.701		0.832
**(b)**					
	**Helmet**		**N**	**Mean Rank**	**Sum of Ranks**
Injury Severity Score (ISS)	Required— Not Worn		190	128.61	24,435.50
	Required—Worn		71	137.40	9755.50
	Total		261		
ED/HOSPITAL Initial GCS Total	Required— Not Worn		189	134.12	25,348.50
	Required—Worn		71	120.87	8581.50
	Total		260		
AIS Head	Required— Not Worn		190	128.58	24,429.50
	Required—Worn		71	137.49	9761.50
	Total		261		
	**Injury Severity Score (ISS)**		**ED/HOSPITAL Initial GCS Total**		**AIS Head**
Mann–Whitney U	629.500		6025.500		6284.500
Wilcoxon W	24,435.500		8581.500		24,429.500
Z	−0.841		−1.421		−0.947
Asymp. Sig. (2-tailed)	0.401		0.155		0.344
**(c)**					
**Helmet**	**Died**		**Survived**		**Total**
Required—Not Worn	21		169		190
Required—Worn	10		61		71
Total	31		230		261
	**Value**	**df**	**Asymptotic Significance (2-sided)**	**Exact Sig. (2-sided)**	**Exact Sig. (1-sided)**
Pearson Chi-Square	0.454 ^a^	1	0.500	0.522	0.316
Continuity Correction	0.210 ^b^	1	0.646		
Likelihood Ratio	0.440	1	0.507	0.522	0.316
Fisher’s Exact Test				0.522	0.316
N of Valid Cases	261				
^a^. 0 cells (0.0%) have an expected count less than 5. The minimum expected count is 8.43. ^b^. Computed only for a 2 × 2 table.					
				**Monte Carlo Significance**	
	**Value**	**Approximate Significance**	**Significance**	**99% Confidence Interval Lower Bound**	**99% Confidence Interval** **Upper Bound**
Phi	−0.042	0.500	0.527 ^c^	0.514	0.540
Cramer’s V	0.042	0.500	0.527 ^c^	0.514	0.540
N of Valid Cases	261				
^c^. Based on 10,000 sampled tables with starting seed 334431365.					

**Table 5:** (**a**) Kruskal–Wallis analysis: ETOH level and trauma scores. (**b**) Kruskal–Wallis analysis: ETOH level and length of stay. (**c**) Chi-square analysis: ETOH level and mortality.

**(a)**						
	**Null Hypothesis**	**Test**			**Sig. ^ab^**	**Decision**
1	The distribution of ISS is the same across categories of ETOH_Group.	Independent-Samples Kruskal–Wallis Test			0.079	Retain the null hypothesis.
2	The distribution of AIS_Head is the same across categories of ETOH_Group.	Independent-Samples Kruskal–Wallis Test			0.165	Retain the null hypothesis.
3	The distribution of GCS_Total is the same across categories of ETOH_Group.	Independent-Samples Kruskal–Wallis Test			<0.001	Reject the null hypothesis.
^a^. The significance level is 0.050. ^b^. Asymptotic significance is displayed.						
**Sample 1–Sample 2**	**Test Statistic**	**Std. Error**	**Std. Test Statistic**		**Sig.**	**Adj. Sig.** ^a^
Extreme (≥300)–Low (1–79)	−44.231	38.583	−1.146		0.252	1.000
Extreme (≥300)–High (200–299)	−64.550	30.649	−2.106		0.035	0.352
Extreme (≥300)–Moderate (80–199)	−99.006	30.162	−3.283		0.001	0.010
Extreme (≥300)–Undetectable	−107.316	27.215	−3.943		<0.001	0.001
Low (1–79)–High (200–299)	20.319	32.610	0.623		0.533	1.000
Low (1–79)–Moderate (80–199)	−54.776	32.152	−1.704		0.088	0.885
Low (1–79)–Undetectable	−63.086	29.406	−2.145		0.032	0.319
High (200–299)–Moderate (80–199)	−34.457	22.012	−1.565		0.117	1.000
High (200–299)–Undetectable	−42.767	17.761	−2.408		0.016	0.160
Moderate (80–199)–Undetectable	−8.310	16.905	−0.492		0.623	1.000
Each row tests the null hypothesis that the Sample 1 and Sample 2 distributions are the same. Asymptotic significances (two-sided tests) are displayed. The significance level is 0.050. ^a^. Significance values have been adjusted by the Bonferroni correction for multiple tests.						
**(b)**						
	**Null Hypothesis**	**Test**			**Sig. ^ab^**	**Decision**
1	The distribution of ED_LOS is the same across categories of ETOH_Group.	Independent-Samples Kruskal–Wallis Test			0.224	Retain the null hypothesis.
2	The distribution of ICU_LOS is the same across categories of ETOH_Group.	Independent-Samples Kruskal–Wallis Test			0.249	Retain the null hypothesis.
3	The distribution of HOSPITAL_LOS is the same across categories of ETOH_Group.	Independent-Samples Kruskal–Wallis Test			0.102	Retain the null hypothesis.
^a^. The significance level is 0.050. ^b^. Asymptotic significance is displayed.						
**(c)**						
	**Died**		**Survived**		**Total**	
ETOH_Group Extreme (≥300)	6		20		26	
High (200–299)	8		60		68	
Low (1–79)	8		14		22	
Moderate (80–199)	7		72		79	
Undetectable	35		269		304	
Total	64		435		499	
	**Value**	**df**	**Asymptotic Significance (2-sided)**	**Monte Carlo Sig. (2-sided) Significance**	**Monte Carlo Sig. (2-sided) 99% Confidence Interval Lower Bound**	**Monte Carlo Sig. (2-sided) 99% Confidence Interval Upper Bound**
Pearson Chi-Square ^a^	14.993	4	0.005	0.007 ^b^	0.005	0.009
Likelihood Ratio	11.686	4	0.020	0.026 ^b^	0.022	0.030
Fisher–Freeman–Halton Exact Test	12.486			0.013 ^b^	0.010	0.016
N of Valid Cases	499					
^a^. 2 cells (20.0%) have an expected count less than 5. The minimum expected count is 2.82. ^b^. Based on 10,000 sampled tables with starting seed 1502173562.						
	**Value**	**Approximate Significance**	**Monte Carlo Significance**		**Monte Carlo Significance 99% Confidence Interval Lower Bound**	**Monte Carlo Significance 99% Confidence Interval Upper Bound**
Phi	0.173	0.005	0.007 ^c^		0.005	0.009
Cramer’s V	0.173	0.005	0.007 ^c^		0.005	0.009
N of Valid Cases	499					
^c^. Based on 10,000 sampled tables with starting seed 1502173562.						

**Table 6:** (**a**) Chi-square analysis of age and mortality. (**b**) Chi-square analysis of race and mortality.

**(a)**						
**Age_Group**	**Died**		**Survived**			**Total**
0 < age < 15	5		17			22
15 ≤ age ≤ 23	13		80			93
24 ≤ age ≤ 64	65		329			394
age ≥ 65	32		62			94
Total	115		488			603
	**Value**	**df**	**Asymptotic Significance (2-sided)**	**Monte Carlo Sig. (2-sided) Significance**	**Monte Carlo Sig. (2-sided) 99% Confidence Interval Lower Bound**	**Monte Carlo Sig. (2-sided) 99% Confidence Interval Upper Bound**
Pearson Chi-Square ^a^	17.095 ^a^	3	<0.001	<0.001 ^b^	<0.001	0.001
Likelihood Ratio	15.341	3	0.002	0.001 ^b^	<0.001	0.002
Fisher–Freeman–Halton Exact Test	15.538			0.001 ^b^	<0.001	0.002
N of Valid Cases	603					
^a^. 1 cell (12.5%) has an expected count less than 5. The minimum expected count is 4.20. ^b^. Based on 10,000 sampled tables with starting seed 329836257.						
	**Value**	**Approximate Significance**	**Monte Carlo Significance**	**Monte Carlo Significance 99% Confidence Interval Lower Bound**	**Monte Carlo Significance 99% Confidence Interval Upper Bound**	
Phi	0.168	<0.001	<0.001 ^c^	<0.001	0.001	
Cramer’s V	0.168	<0.001	<0.001 ^c^	<0.001	0.001	
N of Valid Cases	603					
^c^. Based on 10,000 sampled tables with starting seed 329836257.						
**(b)**						
**Race**	**Died**		**Survived**			**Total**
Asian	22		56			78
Black	3		24			27
Native Hawaiian or Other Pacific Islander	2		2			4
Other	59		315			374
Unknown	9		24			33
White	20		67			87
Total	115		488			603
	**Value**	**df**	**Asymptotic Significance (2-sided)**	**Monte Carlo Sig. (2-sided) Significance**	**Monte Carlo Sig. (2-sided) 99% Confidence Interval Lower Bound**	**Monte Carlo Sig. (2-sided) 99% Confidence Interval Upper Bound**
Pearson Chi-Square ^a^	12.739 ^a^	5	0.026	0.028 ^b^	0.024	0.032
Likelihood Ratio	11.891	5	0.036	0.038 ^b^	0.033	0.043
Fisher–Freeman–Halton Exact Test	12.655			0.023 ^b^	0.019	0.026
N of Valid Cases	603					
^a^. 2 cells (16.7%) have an expected count less than 5. The minimum expected count is 0.76. ^b^. Based on 10,000 sampled tables with starting seed 329836257.						
	**Value**	**Approximate Significance**	**Monte Carlo Significance**	**Monte Carlo Significance 99% Confidence Interval** **Lower Bound**	**Monte Carlo Significance 99% Confidence Interval** **Upper Bound**	
Phi	0.145	0.026	0.028 ^c^	0.024	0.032	
Cramer’s V	0.145	0.026	0.028 ^c^	0.024	0.032	
N of Valid Cases	603					
^c^. Based on 10,000 sampled tables with starting seed 329836257.						

**Table 7:** Correlation between age groups and length of stay in the ED, ICU, and hospital overall using Kruskal–Wallis analysis.

	Null Hypothesis	Test	Sig. ^ab^	Decision
1	The distribution of ED length of stay is the same across categories of age group.	Independent-Samples Kruskal–Wallis Test	0.013	Reject the null hypothesis.
2	The distribution of ICU length of stay is the same across categories of age group.	Independent-Samples Kruskal–Wallis Test	<0.001	Reject the null hypothesis.
3	The distribution of hospital length of stay is the same across categories of age group.	Independent-Samples Kruskal–Wallis Test	<0.001	Reject the null hypothesis.

^a^. The significance level is 0.050. ^b^. Asymptotic significance is displayed.

**Table 8:** Examination of AIS head scores and hospital discharge dispositions using Kruskal–Wallis analysis.

	Null Hypothesis	Test		Sig. ^ab^	Decision
1	The distribution of AIS head is the same across categories of hospital discharge disposition.	Independent-Samples Kruskal–Wallis Test		<0.001	Reject the null hypothesis.
^a^. The significance level is 0.050. ^b^. Asymptotic significance is displayed.					
**Sample 1–Sample 2**	**Test Statistic**	**Std. Error**	**Std. Test Statistic**	**Sig.**	**Adj. Sig.** ^a^
Routine Discharge—Other	32.787	22.037	1.488	0.137	1.000
Routine Discharge—Home with services	76.363	36.775	2.076	0.038	0.378
Routine Discharge—Rehabilitation Required	100.232	18.483	5.423	<0.001	0.000
Routine Discharge—Died	152.585	17.416	8.761	<0.001	0.000
Other—Home with services	43.576	40.903	1.065	0.287	1.000
Other—Rehabilitation Required	−67.446	25.736	−2.621	0.009	0.088
Other—Died	119.798	24.980	4.796	<0.001	0.000
Home with services—Rehabilitation Required	−23.869	39.104	−0.610	0.542	1.000
Home with services—Died	76.222	38.611	1.974	0.048	0.484
Rehabilitation Required—Died	52.352	21.910	2.389	0.017	0.169
Each row tests the null hypothesis that the Sample 1 and Sample 2 distributions are the same. Asymptotic significances (2-sided tests) are displayed. The significance level is 0.050. ^a^. Significance values have been adjusted by the Bonferroni correction for multiple tests.					

**Table 9:** Count of primary payor source by hospital discharge disposition.

Primary Payor Source	Died	Home with Services	Other	Rehabilitation Required	Routine Discharge	Total
Auto	1	1	1	5	8	16
Blue Cross	1	1	0	3	7	12
Commercial	14	4	11	18	57	104
Corrections	1	0	1	0	0	2
HMO	0	0	0	0	1	1
Managed Care	0	0	1	1	2	4
Medicaid	19	2	15	26	99	161
Medicare	7	1	1	5	15	29
No Fault	16	8	16	25	46	111
None	18	1	5	1	31	56
Oth Govmt	0	0	2	0	1	3
Other	17	1	3	9	22	52
Self-Pay	7	0	2	1	13	23
Unknown	14	1	4	1	2	22
Workers Comp	0	0	0	3	4	7
Total	115	20	62	98	308	603

**Table 10:** Chi-square analysis of payor source and hospital discharge disposition.

	Value	df	Asymptotic Significance (2-Sided)	Monte Carlo Sig. (2-Sided) Significance	Monte Carlo Sig. (2-Sided) 99% Confidence Interval Lower Bound	Monte Carlo Sig. (2-Sided) 99% Confidence Interval Upper Bound
Pearson Chi-Square	125.990 ^a^	60	<0.001	<0.001 ^b^	<0.001	0.001
Likelihood Ratio	121.544	60	<0.001	<0.001 ^b^	<0.001	<0.001
Fisher–Freeman–Halton Exact Test	118.036			<0.001 ^b^	<0.001	<0.001
N of Valid Cases	603					
^a^. 53 cells (66.3%) have an expected count less than 5. The minimum expected count is 0.03. ^b^. Based on 10,000 sampled tables with starting seed 1451419960.						
**Nominal by Nominal**	**Value**		**Approximate Significance**	**Monte Carlo Significance**	**Monte Carlo Significance 99% Confidence Interval Lower Bound**	**Monte Carlo Significance 99% Confidence Interval Upper Bound**
Phi	0.457		<0.001	<0.001 ^c^	<0.001	0.001
Cramer’s V	0.228		<0.001	<0.001 ^c^	<0.001	0.001
N of Valid Cases	603					
^c^. Based on 10,000 sampled tables with starting seed 1451419960.						

## Data Availability

The data were requested from the Elmhurst Trauma Registry and extracted using electronic medical records after receiving approval from the Institutional Review Board at our facility (Elmhurst Hospital Center).
